# Timing dependent neuronal migration is regulated by Cdk5-mediated phosphorylation of JIP1

**DOI:** 10.3389/fcell.2024.1371568

**Published:** 2024-03-28

**Authors:** Qinglin Fei, Doo Soon Im, Yiwen Xu, Tianwen Huang, Dianbo Qu

**Affiliations:** ^1^ Department of Pancreatic Surgery, Fudan University Shanghai Cancer Center, Shanghai, China; ^2^ Department of Oncology, Shanghai Medical College, Fudan University, Shanghai, China; ^3^ Shanghai Pancreatic Cancer Institute, Shanghai, China; ^4^ Pancreatic Cancer Institute, Fudan University, Shanghai, China; ^5^ Department of Clinical Neurosciences, Hotchkiss Brain Institute, Cumming School of Medicine, University of Calgary, Calgary, AB, Canada; ^6^ Fujian Key Laboratory of Vascular Aging, Department of Neurology, Fujian Medical University Union Hospital, Fuzhou, China; ^7^ Department of Cellular and Molecular Medicine, Faculty of Medicine, University of Ottawa, Ottawa, ON, Canada

**Keywords:** neuromigrationneuronal migration, CDK5, JIP1, T205, cortical development

## Abstract

The mammalian brain, especially the cerebral cortex, has evolved to increase in size and complexity. The proper development of the cerebral cortex requires the coordination of several events, such as differentiation and migration, that are essential for forming a precise six-layered structure. We have previously reported that Cdk5-mediated phosphorylation of JIP1 at T205 modulates axonal out-growth. However, the spatiotemporal expression patterns and functions of these three genes (Cdk5, Cdk5r1 or p35, and Mapk8ip1 or JIP1) in distinct cell types during cortical development remain unclear. In this study, we analyzed single-cell RNA-sequencing data of mouse embryonic cortex and discovered that Cdk5, p35, and JIP1 are dynamically expressed in intermediate progenitors (IPs). Pseudotime analysis revealed that the expression of these three genes was concomitantly upregulated in IPs during neuronal migration and differentiation. By manipulating the expression of JIP1 and phospho-mimetic JIP1 using *in utero* electroporation, we showed that phosphorylated JIP1 at T205 affected the temporal migration of neurons.

## 1 Introduction

The expansion and differentiation of the cerebral cortex, an evolutionarily advanced structure, have played a significant role in the increase in size and complexity of primate brains (Ohtaka-Maruyama and Okado, 2015; Galakhova et al., 2022). The cerebral cortex undergoes a complex process of development, characterized by a sequence of temporally and spatially coordinated events, such as proliferation, differentiation, migration and synaptogenesis. In particular, the highly coordinated migration of neurons is essential for setting a precise six layered architecture. Due to the intricate nature of temporospatial corticogenesis, which is regulated by numerous intrinsic and extrinsic factors, any disruptions occurring during this crucial process can result in cortical malformations. These malformations are closely linked to various cognitive impairments, ranging from mild to severe intellectual disabilities and autism (Guerrini and Dobyns, 2014). Accordingly, the cortical malformations provide valuable in-sights into the understanding of normal cortical development in the mammalian brain. Since cortical projection neurons are generated directly from radial glial cells in the ventricular zone and indirectly from intermediate progenitor cells in the subventricular zone, then migrate along radial fibers to form the highly organized cortical layers, their dynamic migration and differentiation offer us an opportunity to explore gene’s function by artificially manipulating expression of genes in progenitor or newborn neurons (Kriegstein and Alvarez-Buylla, 2009; Lui et al., 2011; Taverna et al., 2014; Montiel et al., 2016). It is well established that neuronal migration contributes to the regulation of connectivity and activity in developing networks through neurite outgrowth. The rate of axonal outgrowth plays a crucial role in neuronal differentiation, ultimately influencing neuronal activity. Precocious or serotinous interaction between axon and substrate a significantly impact brain connectivity (Halloran and Kalil, 1994; Mouveroux et al., 2000; Mire et al., 2012). Various molecular players that affect the speed of axon growth have been identified (Fenlon, 2022). Although the JNK-interacting protein 1 (JIP1), one of them, has been demonstrated that its phosphorylation by cyclin-dependent kinase 5(Cdk5)/p35 affects the speed of axon growth (Im et al., 2022), the outcome of regulating neuronal migration on architecture of cerebral cortex remains to be fully elucidated. The mammalian neocortex has a similar structure in rodents and primates, consisting of six layers of neurons that migrate from inner to outer regions during development. This allows us to use the mouse model to study how genes potentially affect human brain features and disorders. In this study, we examine how Cdk5 regulates JIP1 phosphorylation and its role in neuronal migration.

## 2 Materials and methods

All animal procedures conducted in this study received approval from both the University of Ottawa Animal Care Committee and the University of Calgary Animal Care Committee. The animals were carefully maintained in full compliance with the Guidelines for the Use and Treatment of Animals established by the Animal Care Council of Canada, which are endorsed by the Canadian Institutes of Health Research.

### 2.1 Antibodies

Mouse anti-JIP1 (B-7, Cat. #sc-25267), Rabbit anti-p35 (C-19, Cat. #sc-820), Rabbit anti-Cdk5 Antibody (C-8, Cat. # sc-173) were purchased from Santa Cruz Biotechnology (Texas, United States). Mouse anti-GFP (9F.F9, Cat. #ab1218) was purchased from abcam (Cambridge, UK). Mouse monoclonal anti-β-actin (Cat. #A5316) was purchased from Sigma-Aldrich (Missouri, United States).

### 2.2 Animals

P35-deficient mice and JIP1-deficient mice were described previously (Im et al., 2022).

### 2.3 In utero electroporation

*In utero* electroporation was performed as previously described (Im et al., 2022). In brief, embryos of pregnant female mice at E14.5 were used for electroporation. A plasmid containing JIP1 or its mutants, along with GFP, under the control of a separate pCGI2 promoter, was injected into the lateral ventricle of the embryonic brain using an Eppendorf Femtojet Microinjector. Subsequently, electroporation was performed in the dorsolateral cortex using a BTX ECM 830 Square Wave Electroporator. Animals were sacrificed as described in figure legend. For analysis, electroporated sections at equivalent rostro-caudal levels of the brain, relative to the corpus callosum and anterior commissure, were selected.

### 2.4 Western blot

Immunoblotting was performed as previously described. Briefly, Protein quantification was performed using the traditional Bradford method. The protein samples were separated by electrophoresis on a 10% polyacrylamide gel in Tris-Glycine/SDS buffer and subsequently transferred onto a polyvinylidene difluoride membrane (PVDF) for immunoblotting. The membranes were then blocked using 5% bovine serum albumin (BSA). The target proteins, including mouse anti-JIP1 (diluted 1:1,000), rabbit anti-p35 (diluted 1:1,000), Cdk5 (diluted 1:1,000), and Mouse anti-β-actin (1:40,000), were probed on the membranes. Following an overnight incubation, the membranes were washed with TBST (Tris-buffered saline with Tween). Subsequently, the membranes were incubated with the corresponding horseradish peroxidase (HRP)-conjugated secondary antibodies (1:10,000). Finally, the Western blot signals were detected using an enhanced chemiluminescence (ECL) method. (Thermo Fisher, Cat. # 32106 or EMD Millipore, Cat. #WBKLS0500).

### 2.5 Software for analysis

Data from mouse perspective somatosensory cortex tissue at E12, E14, E16, and E18 was obtained from the Gene Expression Omnibus (GEO) with accession code GSE153164. To identify variable genes across clusters, the Seurat R package (Di Bella et al., 2021) was applied as previously described. The Monocle3 was used to infer trajectories and show the dynamic changes of Cdk5, Cdk5r1 and Mapk8ip1 genes along the pseudotime. The t-SNE (Rtsnev1.4 package, https://github.com/lmweber/Rtsne-example) plotting was used to show transcript relationships between clusters.

### 2.6 Statistical analysis

All statistical analyses were employed by using GraphPad Prism software version 8.0 (RRID: SCR_002798). All data are presented as the mean ± SEM. To compare multiple groups, either one-way or two-way analysis of variance (ANOVA) was conducted. Post hoc comparisons following ANOVA were performed using the Tukey test. For comparing two groups, an unpaired two-tailed Student's t-test was employed.

## 3 Results and discussion

**FIGURE 1:**
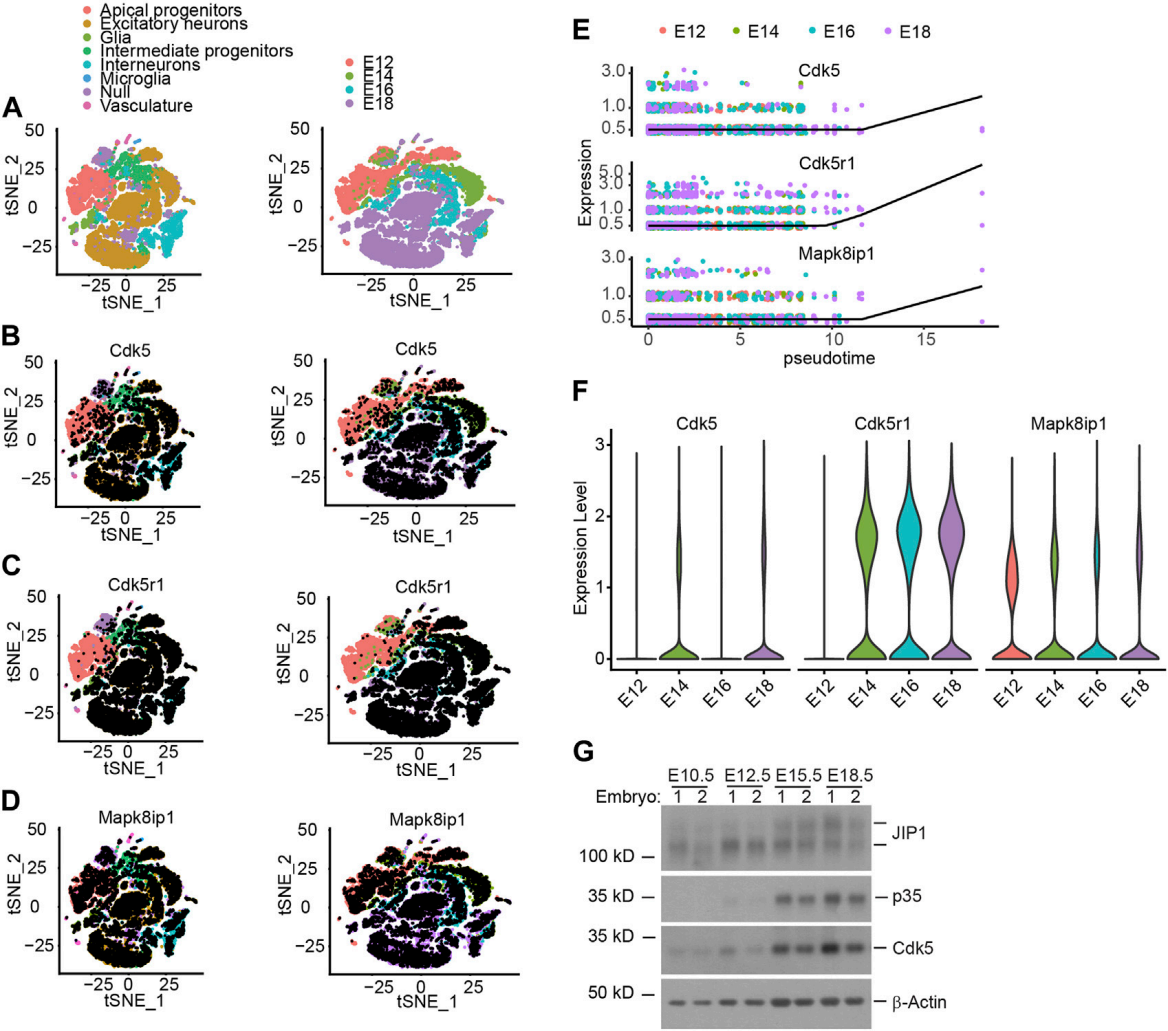
The relationship between expression of Cdk5, p35 and JIP1 across cerebral cortex cells. **(A–D)** Low-dimensional representation of single cells from mouse cerebral cortex, based on tSNE embedding of single-cell RNA-seq data 15. Cells are colored based on **(A)** the cell types and animal age, which ranges from embryonic E12 to E18, **(B)** Cdk5 expression, **(C)** Cdk5r1 (p35) expression, **(D)** Mapk8ip1 (JIP1) expression, **(E)** Pseudotime analysis of Cdk5, Cdk5r1 and Mapk8ip1 dynamic expression in Intermediate progenitors. **(F)** Expression of Cdk5, Cdk5r1 and Mapk8ip1 at different embryonic development ages. **(G)** Expression of Cdk5, p35 and JIP1 in embryonic brains was analyzed by Western blotting.

Unlike other Cyclin-dependent kinase (Cdk) family members, Cdk5 is activated by a non-cyclin protein p35 subunit lacking a conserved domain found in cyclins (Ishiguro et al., 1994; Lew et al., 1994; Tsai et al., 1994). Notwithstanding the temporospatial overlap of expression of Cdk5 and p35 transcripts in the developing mouse neocortex, the expression of p35 is predominantly detected in the post-mitotic neurons (Tsai et al., 1994). We obtained the scRNA-seq data (Di Bella et al., 2021) from the database and performed t-SNE dimensionality reduction to analyze the transcriptional profiles of the cells. The t-SNE plot showed that the cells clustered according to their cell types and collection time, corresponding essentially to (i) Apical progenitors (APs), (ii) Excitatory neurons (ENs), (iii) Glia, (iv) Intermediate Progenitors (IPs), (v) interneurons, (vi) Microglia, (vii) Null and (viii) Vasculature, as indicated by the combined expression of cell type–specific markers and embryonic ages (Figure 1A). The analysis further revealed that Cdk5 exhibits ubiquitous expression across all cell types starting from E12 onwards (Figure 1B; Supplementary Figure S1A). In partial contrast, Cdk5r1 (Cyclin Dependent Kinase 5 Regulatory Subunit 1), the gene encoding p35, showed predominate expression in Ens, IPs and interneurons from E14 onwards (Figure 1C; Supplementary Figure S1A), the neuronal linage, consistent with previously reported (Ximerakis et al., 2019) and Mapk8ip1, the gene encoding JIP1, was transcriptionally expressed in almost all cell types from E12 onwards (Figure 1D; Supplementary Figure S1A). As a computational approach, pseudotime analysis can reconstruct the temporal order of biological events from the dynamic expression of genes to study cell development progression (Magwene et al., 2003). Given that scRNA-seq allows for the simultaneous, unbiased analysis of every type of cell at every developmental age in one experiment, we performed pseudotime analysis on the dynamic expression of Cdk5, Cdk5r1 and Mapk8ip1 in ENs, IPs and interneurons. The pseudotime analysis reveals an equally dynamic gene expression of three genes in the three cell types during cortex development (Figure 1E; Supplementary Figure S1B, C), especially a simultaneously increased expression of three genes in IPs (Figure 1E), indicating that the increased expression of three genes in the IPs may involve in regulating neuronal migration and differentiation. To examine the potential correlation between the increased expression of the three genes and the second wave of neuronal migration during embryonic age E14 in mice, we assessed their transcription levels of three genes, Cdk5, Cdk5r1 and Mapk8ip1, at each embryonic age (Figures 1B–F; Supplementary Figure S1A–C). Consistent with previous studies (Chae et al., 1997), the expression of Cdk5, Cdk5r1, and Mapk8ip1 showed a temporal increase during mid-embryonic age E14, aligning with the critical role of Cdk5/p35 kinase in the subsequent wave of neuronal migration following the first wave migration of neurons out of the ventricular zone (Chae et al., 1997; Ohshima et al., 2007; Nishimura et al., 2014; Ye et al., 2014). To gain insight into the dynamic gene expression patterns and their involvement in neurogenesis and neuronal migration during development, we analyzed the expression of Cdk5, p35, and JIP1 at various embryonic stages using Western blotting. This analysis allowed us to characterize the highly correlated expression dynamics that reflect the overlapping temporal and spatial functions of these genes. Cdk5, p35, and JIP1 were found to be stably expressed from E14 onwards (Figure 1G), which agrees with scRNA-seq data. On the other hand, the deficiency of p35 had no effect on the level of JIP1, and *vice versa* (Supplementary Figure S1D). Taken together, our data suggests the correlated increase in expression of three genes from E12 onwards.

**FIGURE 2:**
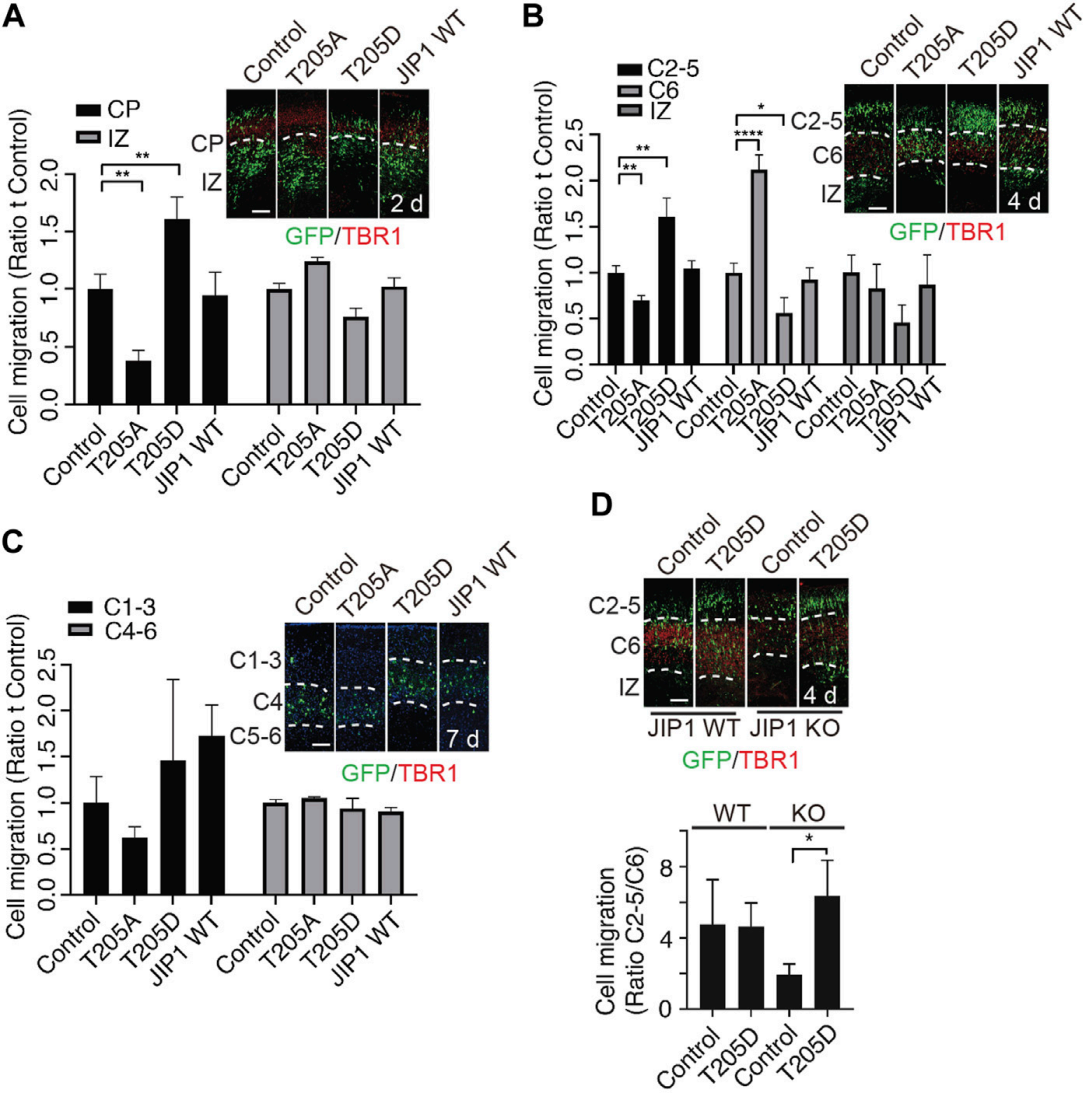
Effects of phospho-JIP1 on cell migration. **(A–C)** CD-1 mice were subjected to *in utero* electroporation (IUE) with GFP control (empty vector), JIP1 (T205A)-IRES-GFP, JIP1 (T205D)-IRES-GFP or JIP1 WT-IRES-GFP at E14.5. The embryonic brain tissues were collected 2, 4 and 7 days after IUE and labeled with green fluorescent protein (GFP) and TBR1. **(A)** The migration of GFP-positive cells was analyzed in the cortical plate (CP) and intermediate zone (IZ) at E16.5 (2 days after IUE). Quantification of cell migration by comparing the number of GFP-positive cells expressing JIP1, JIP1(T205A) or JIP1(T205D) residing at CP or IZ to the number of controls only expressing GFP at E16.5 was analyzed by two-way ANOVA. Data presented as mean ± SEM; n = 7 animals for each group. **(B)** The migration of GFP-positive cells expressing JIP1, JIP1(T205A) or JIP1(T205D) was analyzed in cortical layers 2–5 (C2-5), cortical layer 6 (C6), and the IZ at E18.5 (4 days after IUE) by comparing to the controls only expressing GFP. The migration of cells at E18.5 was quantified using an independent sample t-test. The results are presented as the mean ± SEM, with n = 12 animals for the vector group, n = 10 animals for the T205A group, n = 7 animals for the T205D group, and n = 11 animals for the wild-type JIP1 group. **(C)** Representative images of cell migration 7 days after IUE. GFP positive cells were analyzed for the distribution at cortical layer 1–3 (C1-3), cortical layer 4 (C4) and cortical layer 5–6 (C5-6). The migration of cells to the cortical layers 1–3 (C1-3) and 4–6 (C4-6) was quantified using a two-way analysis of variance (ANOVA). Data presented as mean ± SEM; n = 4 animals for vector, n = 4 animals for T205A, n = 3 animals for T205D and n = 3 animals for JIP1 WT. **(D)** JIP1 WT and JIP1 KO mice were subjected to IUE with GFP control (empty vector), and JIP1 (T205D)-IRES-GFP at E 14.5. The embryonic brain tissues were collected 4 days after IUE and labeled with green fluorescent protein (GFP) and TBR1. The number of GFP positive cells in C2-5 was compared to the number of GFP positive cells in C6. The ratio of migrated cells in C2-5 to C6 at E18.5 (4 days after IUE) was analyzed using a two-way ANOVA and unpaired two-tailed Student’s t-test. The results are presented as the mean ± SEM, with JIP1 WT/Het animals (n = 4) for vector and JIP1 WT animals (n = 6) for T205D, JIP KO animals (n = 4) for vector and JIP KO animals (n = 6) for T205D. ‘n’ represents the number of animals. *p < 0.05, **p < 0.01 and ****p < 0.0001. Scale bar = 100 µm.

In our previous study, we showed that Cdk5-mediated phosphorylation of JIP1 at Thr 205 promotes neuroaxonal outgrowth (Im et al., 2022). Here, we conducted an investigation to determine the involvement of Cdk5-mediated phosphorylation of JIP1 at Thr 205 in neuronal migration. According to a large number of growing evidence that altered timing during the neurodevelopment can trigger massive changes in phenotypes, leading to individual variability, as well as malformation and disease (Telley et al., 2019; Delgado et al., 2022) we hypothesize that JIP1 and phosphorylated JIP1 at Thr 205 may regulate the timing of migrating neurons, despite JIP1 KO mice showing no phenotypic outcomes (Whitmarsh et al., 2001). To test this possibility more directly, we performed *in utero* electroporation (IUE) at E14.5, overexpressing wild type (WT) JIP1, non-phosphorylatable JIP1(T205A), phospho-mimetic JIP1(T205D), or GFP control in cortical cells and analyzed their distribution at 2 days, 4 days and 7 days after IUE in CD1 mice (Figures 2A–C). Our findings indicate that the overexpression of JIP1(T205D) in cortical migrating neurons enhanced their migration at E16.5 (2 days after IUE) and E18.5 (4 days after IUE). Conversely, the expression of JIP1(T205A) inhibited neuronal migration. In contrast, both wild-type JIP1 and the GFP control did not demonstrate any significant effects on migration (Figures 2A,B)). Interestingly, all the neurons expressing exogenous genes migrated to their destinated layer, cortical plate 7 days after IUE (Figure 2C). Together, these data suggest that timing dependence is a functional role of phosphorylation of JIP1 at Thr 205 in regulating cortical neuronal migration. Since the effects on neuronal migration were observed when overexpressing JIP1 mutant proteins in WT mice, we performed a set of rescue IUE in JIP1 knockout (KO) embryos at E14.5 by overexpressing JIP1(T205D). Analysis E18.5 (4 days after IUE) (Figure 2D) but not at E16.5 (2 days after IUE) (Supplementary Figure S1E) showed that there existed more migrating neurons when expressing JIP1(T205D) when comparing to controls, suggesting phospho-mimetic mutant JIP1(T205D) rescued the impeded migration caused by JIP1 deficiency. We next validated the role of Cdk5 in neuronal migration regulated by phospho-JIP1 at Thr 205, the JIP1(T205D) mutant was expressed in p35 KO embryos at E14.5 using IUE. The disorganized cortical layered architecture observed in p35 knockout (KO) embryos (Supplementary Figure S1F) aligns with previous findings indicating that neurons generated earlier tend to populate superficial layers, while those generated later tend to localize in deeper layers (Chae et al., 1997; Ko et al., 2001; Ohshima et al., 2007). Unfortunately, this aberrant cortical organization in p35 KO embryos precluded us from conducting a comprehensive analysis of neuronal migration, a comparison we successfully performed in WT and JIP1 KO embryos. Consequently, we were unable to assess whether the introduction of JIP1(T205D) expression in neuronal progenitors could rescue neuronal migration observed in the cortex.

## 4 Conclusion

Nonetheless, given that Cdk5-mediated phosphorylation of JIP1 at Thr 205 promotes neuroaxonal outgrowth via facilitating ITCH-mediated NOTCH1 degradation (Im et al., 2022), our migration results suggest that the Cdk5-JIP1 axis may also play a regulatory role in control of neuronal migration.

## Data Availability

The original contributions presented in the study are included in the article/Supplementary Material, further inquiries can be directed to the corresponding authors.

## Supplementary material

SUPPLEMENTARY FIGURE S1 Expression of Cdk5, p35 and JIP1 in different ages. **(A)** Expression of Cdk5, Cdk5r1 and Mapk8ip1 in different cell types based on single-cell RNA-seq data (2). **(B)** Pseudotime analysis of Cdk5, Cdk5r1 and Mapk8ip1 dynamic expression in excitatory neurons. **(C)** Pseudotime analysis of Cdk5, Cdk5r1 and Mapk8ip1 dynamic expression in interneurons. **(D)** Expression of p35 and JIP1 in wild type, p35 KO and JIP1 KO brains at E15. **(E)** JIP1 WT and JIP1 KO mice were subjected to IUE with GFP control (empty vector), and JIP1 (T205D)-IRES-GFP at E 14.5. The embryonic brain tissues were collected 2 days after IUE and labeled with green fluorescent protein (GFP) and TBR1. **(F)** Embryonic brain tissues from p35 WT and KO mice at E14.5 were subjected to IUE with GFP control vector. 4 days after IUE, the brain tissues were stained using GFP and TBR1 antibodies. Scale bar = 100 µm.

## Abbreviations

JIP1, The JNK-interacting protein 1; Cdk5, Cyclin-dependent kinase 5; CDK5R1, Cyclin dependent kinase 5 regulatory subunit 1; scRNA-seq, single-cell RNA-sequencing; Mapk8ip1, mitogen-activated protein kinase 8 interacting protein 1; APs, Apical progenitors; ENs, Excitatory neurons; IPs, Intermediate Progenitors.
