# Supplementary figures and images for: Timing dependent neuronal migration is regulated by Cdk5-mediated phosphorylation of JIP1
Source: Front Cell Dev Biol. 2024 Mar 28;12:1371568. doi: 10.3389/fcell.2024.1371568 (PMC11007206; doi:10.3389/fcell.2024.1371568)

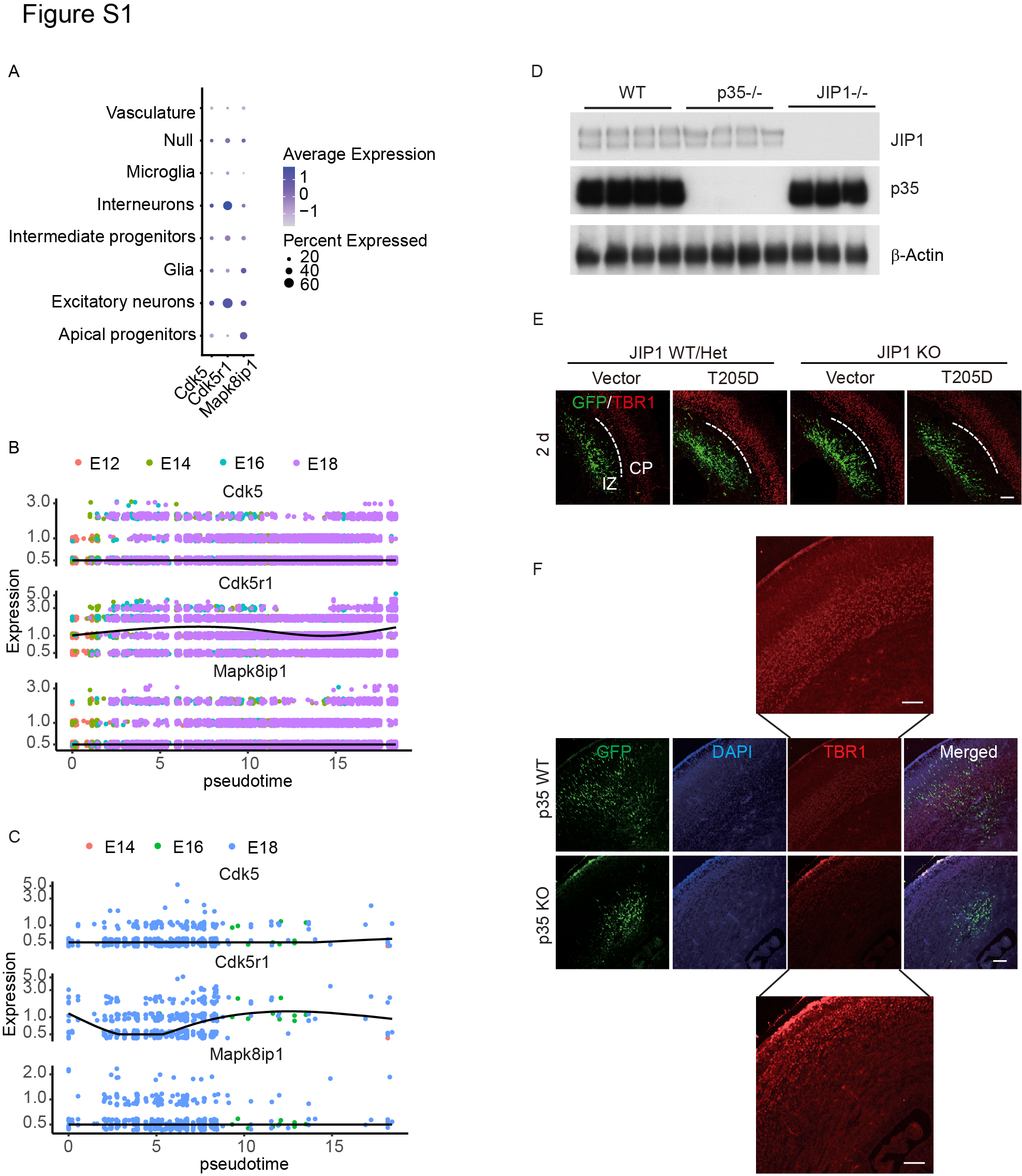

Supplement: Supplementary file 1 [file Image1.jpeg]
